# Supplementary figures and images for: Early IgE Production Is Linked with Extrafollicular B- and T-Cell Activation in Low-Dose Allergy Model
Source: Vaccines (Basel). 2022 Jun 17;10(6):969. doi: 10.3390/vaccines10060969 (PMC9231339; doi:10.3390/vaccines10060969)

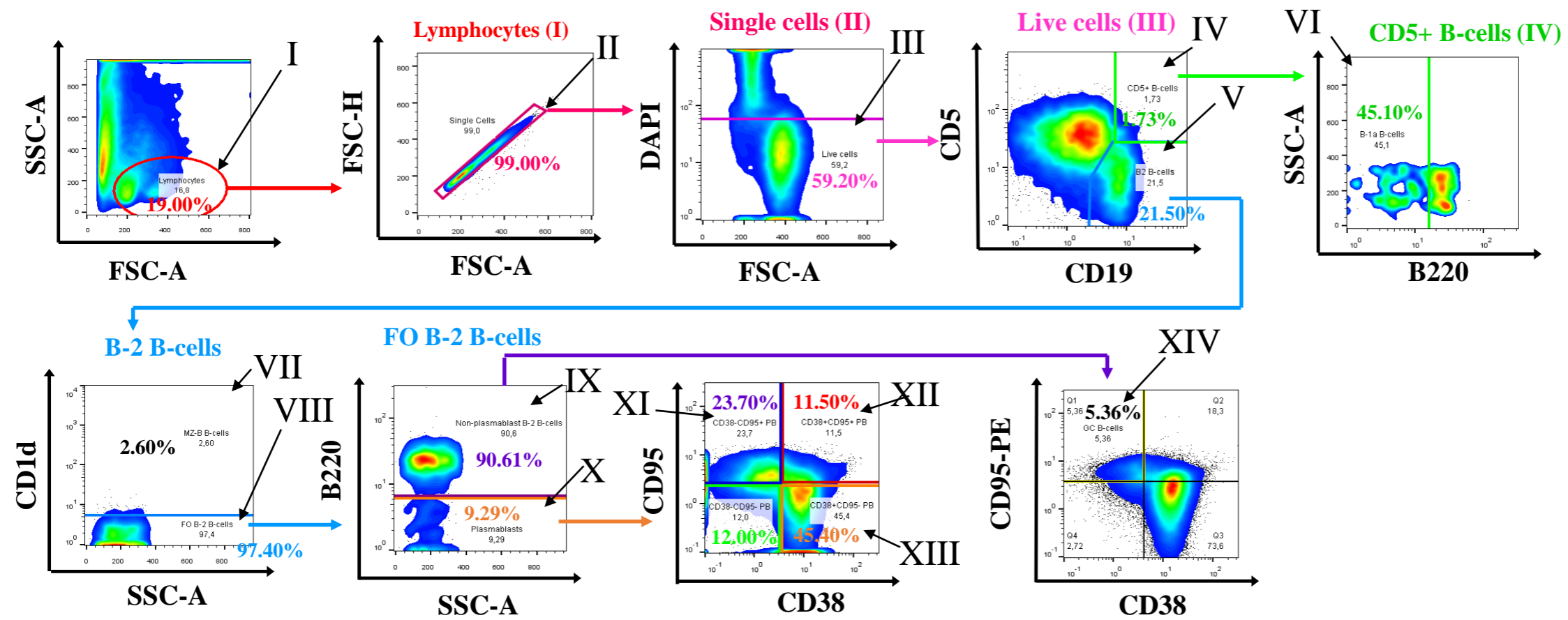

**Figure S1**

Supplement: Supplementary file 1 [file vaccines-10-00969-s001.zip › Figure S1 19-05-22 Chudakov DB.pdf]

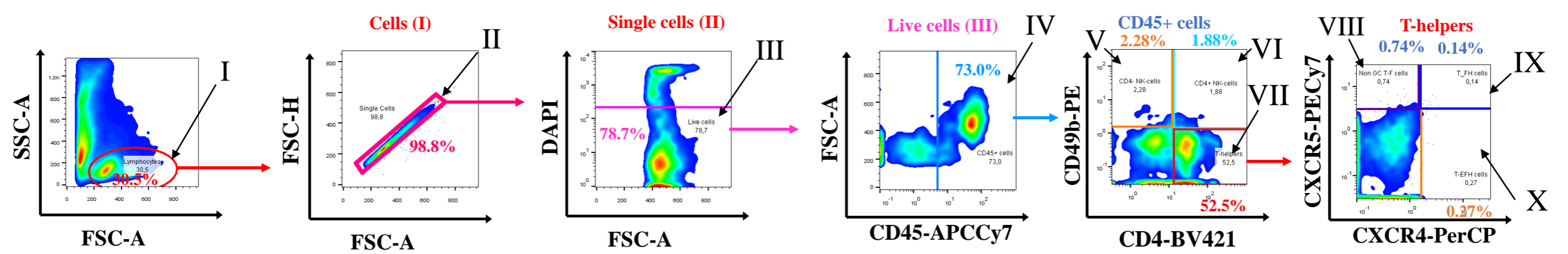

**Figure S2**

Supplement: Supplementary file 1 [file vaccines-10-00969-s001.zip › Figure S2 01-06-22 Chudakov DB.pdf]
